# Differences in Oxidative Stress Markers and Antioxidant Enzyme Activities in Black Bean Aphid Morphs (*Aphis fabae* Scop.) Fed on the Primary Host *Viburnum opulus* L.

**DOI:** 10.3390/antiox11122476

**Published:** 2022-12-16

**Authors:** Iwona Łukasik, Sylwia Goławska, Hubert Sytykiewicz

**Affiliations:** Institute of Biological Sciences, Faculty of Exact and Natural Sciences, Siedlce University of Natural Sciences and Humanities, 08-110 Siedlce, Poland

**Keywords:** black bean aphid, reactive oxygen species, oxidative stress, antioxidant enzymes

## Abstract

Changes in the level of oxidative stress markers—superoxide anion radical ($O_{2^{-}}$), hydrogen peroxide (H_2_O_2_) and malondialdehyde (MDA) and the activity of antioxidant enzymes—superoxide dismutase (SOD), catalase (CAT) and ascorbate peroxidase (APX) in the black bean aphid occurring on the primary host (viburnum plants) were studied. Among the aphid morphs, the lowest contents of $O_{2^{-}}$, H_2_O_2_ and MDA were noted for winged adults (*alatae*), which were also characterized by the highest activity of antioxidant enzymes. These metabolic features indicate the adaptation of winged morphs to the colonization of new host plants. During spring migration, an increase in the content of oxidative stress markers and antioxidant enzyme activities in wingless females (*fundatrigeniae*) was observed. The significance of the biochemical adaptation of the black bean aphid to its winter host is discussed.

## 1. Introduction

Black bean aphid (*Aphis fabae* Scop.) is one of the most agriculturally important pests of crops worldwide [1]. *A. fabae* is a highly polyphagous species that shows host alternation involving seasonal movements between primary and secondary host plants. In the case of *A. fabae*, the primary hosts are *Euonymus europaeus* L., *Viburnum opulus* L. and *Philadelphus coronarius* L. [2,3,4]. *A. fabae* has many secondary hosts, including *Vicia faba* L., *Chenopodium album* L., *Lamium purpureum* L., *Papaver dubium* L., *Beta vulgaris* L., *Capsella bursa-pastoris* L., *Veronica hederifolia* L., *Amaranthus retroflexus* L. and *Capsicum annuum* L. [3,5,6,7]. *A. fabae* overwinters in the egg stage on a winter host. In spring, wingless females (*fundatrices*) develop from winter eggs. In Poland, the first larvae appear in early spring, when the temperature fluctuates around 7–8° [8]. *Fundatrices* give birth to the larvae of the next spring’s generations (*fundatrigeniae*), which develop into wingless and winged females. In late April and early May, winged migrants colonize the secondary host on which the next generations spend the entire season. In autumn, *alatae gynoparae* appear on the summer host and return to the primary host. After returning, *gynoparae* produce *apterous oviparae*, which mate with the males and lay eggs [9].

Aphids reduce crop yields and are among the most serious pests of various crops and ornamental plants. Interactions are observed between plants and herbivores. On the one hand, aphids have devastating effects on plants, and on the other hand, plants affect aphid behavior, physiology and metabolism and, as a result, reduce aphid populations [10,11]. *A. fabae* feeding results in yield losses causing malformations of younger leaves, shoots and flowers. *A. fabae* can also cause indirect damage as the vector for more than 30 plant viruses, including alfalfa mosaic virus, cucumber mosaic virus, lettuce mosaic virus, pepper veinal mottle virus and plum pox virus [7,12,13,14].

One of the primary hosts of *A. fabae* is the cranberry bush *Viburnum opulus* L., commonly known as guelder rose or highbush cranberry, which is one of the most widespread shrub species. The guelder rose is widely cultivated in gardens in many countries in Europe and Asia. The fruit of *V. opulus* is rich in biologically active substances known for its antioxidant properties [15,16,17,18,19]. Phytochemical studies on this species have shown the presence of different natural compounds, including iridoids, iridoid glucosides, lantanoside, flavonoids, saponins, tannins, arbutin, ursolic acid, flavones and anthocyanins [20,21,22].

The penetration of aphid stylets causes mechanical damage that may induce plant defense-signaling pathways based on the generation of reactive oxygen species (ROS), such as superoxide anion radical ($O_{2^{-}}$) and hydrogen peroxide (H_2_O_2_) [23,24]. H_2_O_2_ exhibits direct toxicity towards phytophagous insects, contributes to cell wall strengthening processes, triggers hypersensitive responses (HR) and acts as a signal molecule for the induction of defense genes [25]. Moreover, plants produce pro-oxidant phytochemicals that, upon activation, generate ROS [26]. The overproduction of ROS in host plants, in turn, negatively affects herbivores by evoking oxidative stress in their tissues [27]. The accumulation of ROS cause damage to macromolecules, such as deoxyribonucleic acid (DNA), ribonucleic acid (RNA), proteins, and also lipids. However, herbivorous insects are equipped with antioxidant mechanisms that remove excess ROS. They are composed of low-molecular-weight antioxidants (e.g., glutathione, ascorbate, tocopherol) and antioxidant enzymes. The main enzymatic antioxidants that catalyze the destruction of toxic ROS are superoxide dismutase (SOD) and catalase (CAT). SOD catalyzes the conversion of $O_{2^{-}}$ into oxygen and H_2_O_2_. CAT, presented in the peroxisomes of aerobic cells, protects cells from H_2_O_2_ by catalyzing its disproportionation. Since CAT is inefficient at removing low concentrations of H_2_O_2_, aphids possess an alternative enzyme for their removal—ascorbate peroxidase (APX) [28,29]. APX catalyzes the reduction of H_2_O_2_ with the simultaneous oxidation of ascorbate to dehydroascorbic acid (DHA). Ascorbate is regenerated from DHA by the GSH-dependent enzyme dehydroascorbic acid reductase (DHAR).

To date, little is known about the role of ROS and the antioxidant enzymes of aphids. Little is also known about the adaptation of host-alternating aphids to feeding on the primary host [30,31]. The role of ROS and antioxidant enzymes in the adaptation of *A. fabae* to feeding on *V. opulus* has not been studied. Our study aimed to aid in a better understanding of the role of ROS and antioxidant enzymes in the development of the *A. fabae* population on primary host *V. opulus*. We compared the level of $O_{2^{-}}$, H_2_O_2_ and malondialdehyde (MDA), as well as the activity of antioxidant enzymes (SOD, CAT and APX), in various morphs of *A. fabae* fed on *V. opulus*. We also determined the changes in oxidative stress markers and antioxidant enzyme activities in *fundatrigeniae* tissues during their occurrence in the primary host.

## 2. Materials and Methods

### 2.1. Plants and Aphids

The population of *A. fabae* (*fundatrices, fundatrigeniae* and *alatae)* was monitored on the wild cultivar of its winter host (*V. opulus*) from the third decade of April until the end of June. The number of aphids was determined by directly counting aphid individuals infesting 30 randomly selected, fully expanded, 1-year-old side shoots of *V. opulus*. Entomological observations were conducted once every 10 days during two consecutive vegetation seasons in 2019–2020. The results were reported as the mean number of aphids per side shoot of *V. opulus*. The viburnum trees were located in green areas around Siedlce, Poland (52,012′ N; 22,017′ E).

Samples of aphids for the analysis of biochemical parameters were collected when the monitoring of population density was conducted in four terms: the second decade of May (I), the third decade of May (II), the first decade of June (III), and the second decade of June (IV).

### 2.2. $O_{2^{-}}$ Assay

The superoxide content was assayed following Green and Hill [32], based on the reduction in nitroblue tetrazolium (NBT). Fifty collected aphids were homogenized in 4 mL of 10 mM K-phosphate buffer (pH 7.8) containing a superoxide dismutase inhibitor (1 mM diethyldithiocarbamate). The homogenates were filtered through two layers of cheesecloth and centrifuged at 15,000× *g* for 15 min. The obtained supernatant (0.5 mL) was mixed with 0.5 mL of 0.4 mM nitroblue tetrazolium in 10 mM phosphate buffer (pH 7.8). The increase in absorbance at 490 nm was monitored against the blank containing 0.5 mL of crude homogenate of aphids and 0.5 mL of 0.2 M phosphate buffer (pH 7.8). The NBT-reducing activity of plant extracts was expressed as ΔA_490_ per minute per mg of protein.

### 2.3. H_2_O_2_ Assay

The H_2_O_2_ concentration was estimated following Green and Hill [32]. Twenty aphids were homogenized in 4 mL of 50 mM K-phosphate buffer (pH 7.0) and centrifuged at 15,000× *g* for 15 min. A total of 0.3 mL of supernatant was added to 1 mL of reagent (4 mM 4-aminoantipyrine, 24 mM phenol and 0.4 U/mL peroxidase dissolved in 50 mM phosphate buffer, pH 7.0). The reaction mixture was incubated at 25 °C for 10 min, and absorbance was measured at 510 nm against a blank containing 0.3 mL of distilled water instead of the aphid homogenate. The H_2_O_2_ concentration was calculated from a calibration curve prepared for this standard and expressed in nmol per mg of protein.

### 2.4. MDA Assay

The MDA content was estimated according to Halliwell and Gutteridge [33]. Fifty aphids were homogenized in 6 mL of 0.1% trichloroacetic acid (TCA) and centrifuged at 15,000× *g* for 15 min. The sample contained 1 mL of the aphid homogenate, 1 mL of 0.6% thiobarbituric acid (TBA) in 0.25 M HCl and 1 mL of 15% trichloroacetic acid (TCA). At the same time, two controls were prepared. The first one contained 1 mL of distilled water instead of the aphid homogenate, and in the second one, TBA was replaced with 1 mL of distilled water. The reaction mixtures were placed in a boiling water bath for 60 min. After cooling, the mixtures were centrifuged at 10,000× *g*, and the absorbance of the supernatants was measured at 535 nm against the first control. The value obtained for the second control was subtracted from the absorbance reading for the sample. The MDA concentration was calculated using an extinction coefficient of 156 mM^−1^cm^−1^ [34] and expressed as nmol per mg of protein.

### 2.5. SOD Assay

SOD activity was measured by the method based on its inhibitory action on the rate of autooxidation of pyrogallol [35]. Fifty collected aphids were homogenized in 3 mL of 50 mM Tris-HCl buffer (pH 8.2). The homogenates were filtered through two layers of cheesecloth and centrifuged at 15,000× *g* for 15 min. The reaction mixture was prepared in spectrophotometer cuvettes using 0.90 mL of 50 mM Tris-HCl buffer (pH 8.2), 0.03 mL of 10 µM catalase and 0.045 mL of supernatant. The reaction was started with the addition of 0.025 mL of 30 mM pyrogallol, and the absorbance was measured at 420 nm for 3 min against the control without the enzyme extract. One unit of SOD activity was calculated as the amount of enzyme causing 50% inhibition of pyrogallol autoxidation. SOD activity was expressed as units per milligram of protein.

### 2.6. CAT Assay

CAT activity was measured as described by Aebi [36]. Ten aphids were homogenized in 4 mL of 67 mM K-phosphate buffer (pH 7). The homogenates were filtered through two layers of cheesecloth and centrifuged at 15,000× *g* for 15 min. A total of 0.5 mL of aphid extract was added to 0.5 mL of 30 mM H_2_O_2_, and the disappearance of H_2_O_2_ was measured at 240 nm for 3 min. Catalase activity was expressed as µmol of decomposed H_2_O_2_ per minute per mg of protein.

### 2.7. APX Assay

APX activity was determined by the Asada method [37]. Thirty collected aphids were homogenized in 5 mL of 67 mM K-phosphate buffer pH 7 and centrifuged at 15,000× *g* for 15 min. The sample contained 0.75 mL of aphid extract, 0.25 mL of 67 mM K-phosphate buffer (pH 7) with 2.5 mM ascorbic acid (ASA) and 0.2 mL of 30 mM H_2_O_2_. The decrease in absorbance at 290 nm was monitored for 5 min against the blank containing the boiled sample. The APX activity was expressed as µmol ascorbate oxidized per minute per mg of protein using an extinction coefficient of 2.8 mM^−1^ cm^−1^.

### 2.8. Protein Assay

The protein content in the studied aphid supernatants was determined using the Bradford method [38].

### 2.9. Statistical Analyses

All data were calculated as the mean of the least four independent replicates. Analysis of variance (one-way ANOVA) was performed to examine the significance of the tested variables (content of $O_{2^{-}}$, H_2_O_2_ and MDA, activity of SOD, CAT and APX) in various morphs of *A. fabae* occurring on *V. opulus*, as well as the $O_{2^{-}}$, H_2_O_2_ and MDA content and SOD, CAT and APX activities in *fundatrigeniae* during feeding *A. fabae* on *V. opulus*. Post-hoc Tukey’s test was employed (*p* < 0.05 was set as significant). All statistical analyses were performed using Statistica version 10.0 (Statsoft Inc., Kraków, Poland).

## 3. Results

### 3.1. Population Dynamics of A. fabae on V. opulus

**Figure 1:**
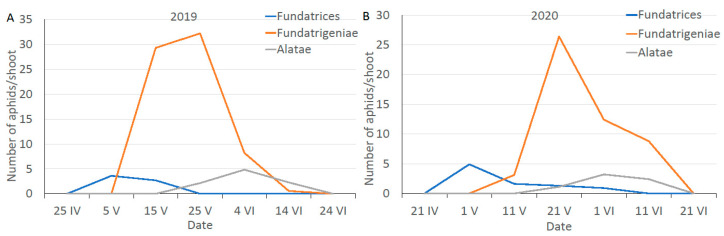
The dynamics of three morphs of the *A. fabae* population on its primary host in the spring periods of 2019 (**A**) and 2020 (**B**).

The first *fundatrices* of *A. fabae*, hatched from winter eggs, were observed on viburnum in the third decade of April. These morphs fed on the opening buds and then on the undersides of young leaves and soon after the first *fundatrigeniae* were found. The density of *fundatrigeniae* on *V. opulus* reached its maximum in the second or third decade of May. At the same time, winged migrants gradually formed. Most left the primary host and colonized summer hosts. Thus, *fundatrigeniae* were still the dominant morphs on viburnum. With the appearance of migrants on *V. opulus*, the *A. fabae* population size began to decline (Figure 1).

### 3.2. $O_{2^{-}}$, H_2_O_2_ and MDA Content in A. fabae Morphs

**Figure 2:**
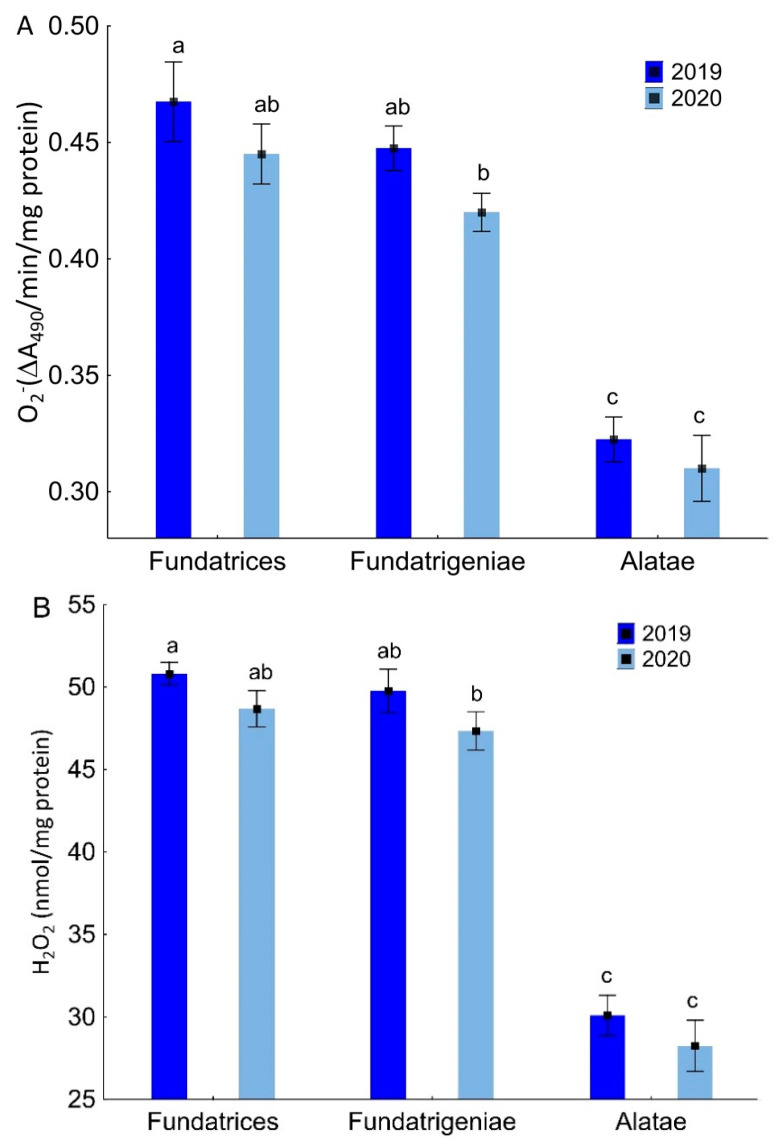

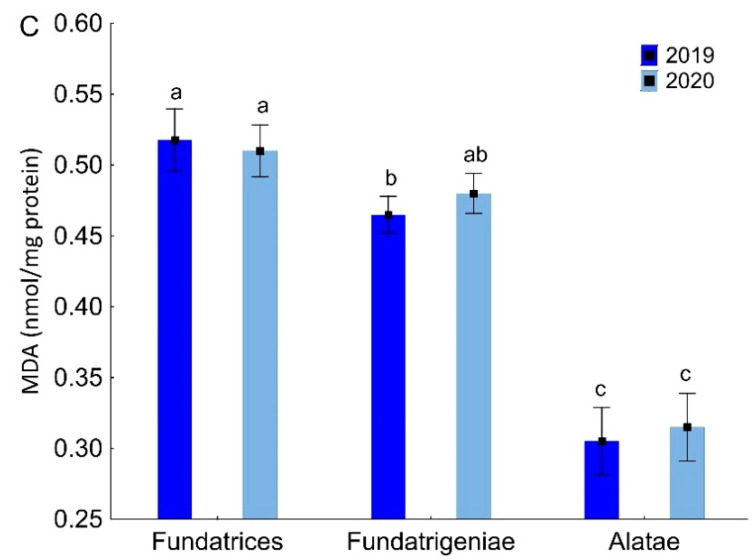
Superoxide anion ($O_{2^{-}}$) (ΔA_490_/min/mg protein) (**A**), hydrogen peroxide (H_2_O_2_) (nmol/mg protein) (**B**) and malondialdehyde (MDA) content (nmol/mg protein) (**C**) in various morphs of *A. fabae* on *V. opulus* (means ± SD; *n* = 4). Different letters denote significant differences (one–way ANOVA; Tukey’s test; *p* < 0.05).

In various morphs of *A. fabae* occurring in *V. opulus*, there were differences in the content of $O_{2^{-}}$ (ANOVA, F_5,18_ = 132.22, *p* < 0.001), H_2_O_2_ (ANOVA, F_5,18_ = 300.34, *p* < 0.001) and MDA (ANOVA, F_5,18_ = 96.22, *p* < 0.001). The lowest contents of the mentioned indicators of oxidative stress were noted in the tissues of winged migrants. *Fundatrices* and *fundatrigeniae* possessed a comparable level of $O_{2^{-}}$, H_2_O_2_ and MDA except for 2019, when *fundatrices* had a higher MDA content. There were no differences in the level of oxidative stress markers between the individual morphs in either year of the study (Figure 2).

### 3.3. SOD, CAT and APX Activity in A. fabae Morphs

**Figure 3:**
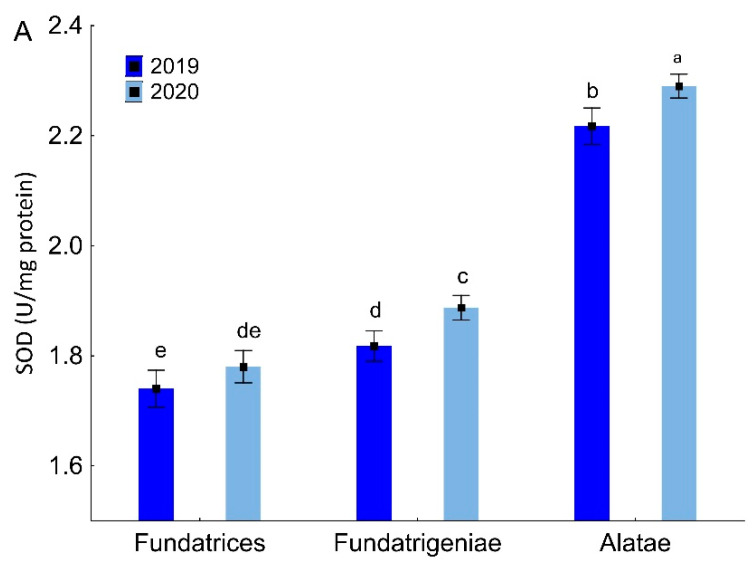

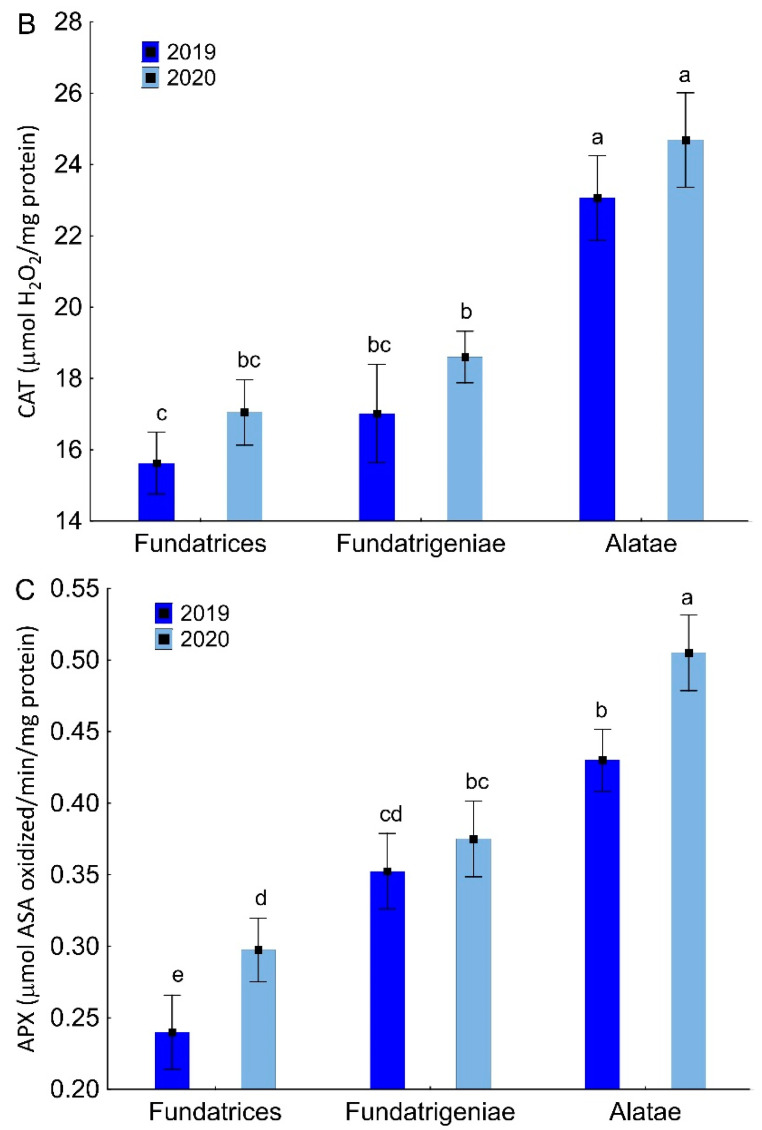
Superoxide dismutase (SOD) (U/mg protein) (**A**), catalase (CAT) (µmol H_2_O_2_/mg protein) (**B**) and ascorbate peroxidase (APX) activity (µmol ASA oxidized/min/mg protein) (**C**) in various morphs of *A. fabae* on *V. opulus* (means ± SD; *n* = 4). Different letters denote significant differences (one–way ANOVA; Tukey’s test; *p* < 0.05).

In various morphs of *A. fabae* occurring in *V. opulus*, there were differences in the content of SOD (ANOVA, F_5,18_ = 281.00, *p* < 0.001), CAT (ANOVA, F_5,18_ = 44.88, *p* < 0.001) and APX (ANOVA, F_5,18_ = 57.14, *p* < 0.001). The highest SOD and APX activities were recorded for *alatae* and the lowest for *fundatrices*. For CAT, wingless *fundatrices* and *fundatrigeniae* were characterized by similar enzyme activity, lower than that of *alatae*. No differences were found in CAT activities in the *A. fabae* morphs in either season of the study. However, the SOD and APX activities for the tested morphs depended on the season. *Fundatrigeniae* and *alatae* exhibited higher SOD activity in 2019, while *fundatrices* had comparable levels of SOD activity in both years of the experiment. Higher APX activities were noted for *fundatrices* and migrants in the first year of the study, in contrast to *fundatrigeniae*, which had similar APX activity in both seasons (Figure 3).

### 3.4. Changes in $O_{2^{-}}$, H_2_O_2_ and MDA Content in the Fundatrigeniae of A. fabae during Their Occurrence on the Primary Host

**Figure 4:**
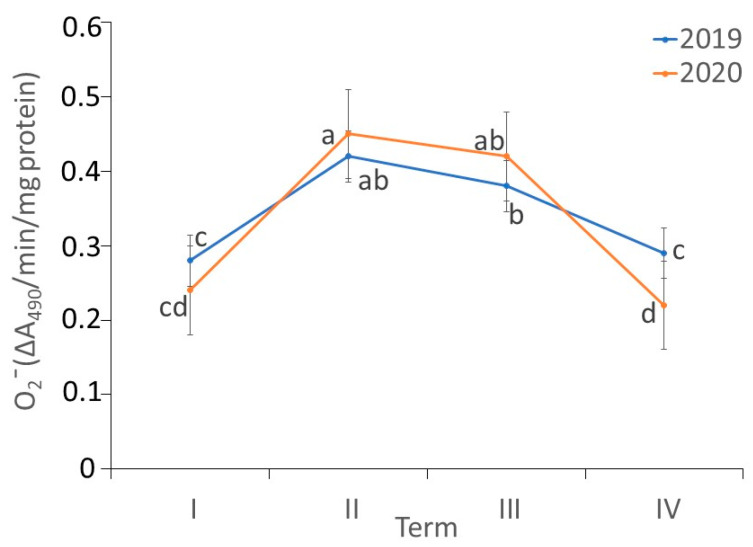
Changes in the superoxide anion radical ($O_{2^{-}}$) content (ΔA_490_/min/mg protein) in *A. fabae fundatrigeniae* during feeding on *V. opulus* (means ± SD; *n* = 4). Different letters denote significant differences (one–way ANOVA; Tukey’s test; *p* < 0.05).

There were differences in the $O_{2^{-}}$ content in the *fundatrigeniae* of *A. fabae* during feeding on *V. opulus* (ANOVA, F_7,24_ = 63.03, *p* < 0.001). In both studied years, the highest level of $O_{2^{-}}$ in *fundatrigeniae* tissues was recorded in the second and third term of observation. The content of $O_{2^{-}}$ in studied morphs was lower in the first and fourth observation periods, but it did not differ in either of these terms. Comparing the years of observation, no differences were noted in the $O_{2^{-}}$ content in the initial period of the occurrence of *fundatrigeniae* on *V. opulus* and during the periods of their largest density on the primary host. At the end of the occurrence of *fundatrigeniae* on *V. opulus*, a higher $O_{2^{-}}$ content was found in 2019 (Figure 4).

**Figure 5:**
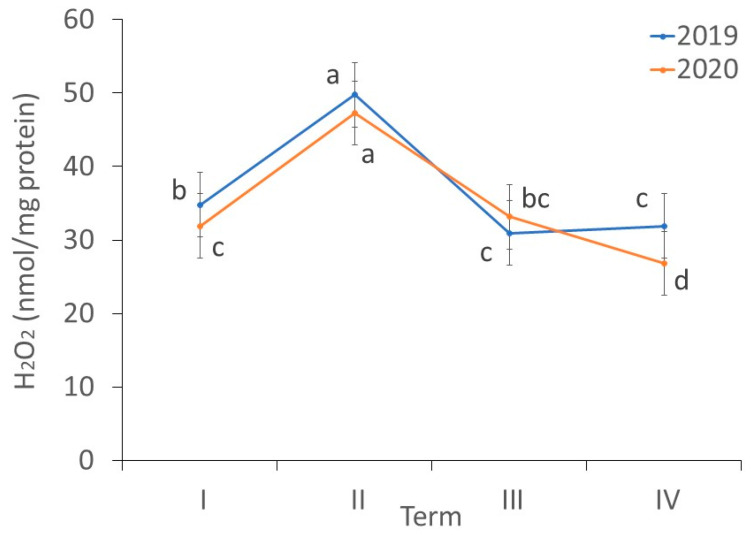
Changes in the hydrogen peroxide (H_2_O_2_) content (nmol/mg protein) in *A. fabae fundatrigeniae* during feeding on *V. opulus* (means ± SD; *n* = 4). Different letters denote significant differences (one–way ANOVA; Tukey’s test; *p* < 0.05).

There were differences in the content of H_2_O_2_ in the *fundatrigeniae* of *A. fabae* during feeding on *V. opulus* (ANOVA, F_7,24_ = 177.13, *p* < 0.001). The highest H_2_O_2_ concentration in the *fundatrigeniae* was noted in the second term of observation. In 2019, the H_2_O_2_ content in wingless morphs was comparable in the third and fourth observation periods, whereas in 2020, the radical content was lowest in the fourth term. In the initial and final parts of aphid occurrence on the primary host, a higher H_2_O_2_ content in *fundatrigeniae* was noted in 2019 (Figure 5).

**Figure 6:**
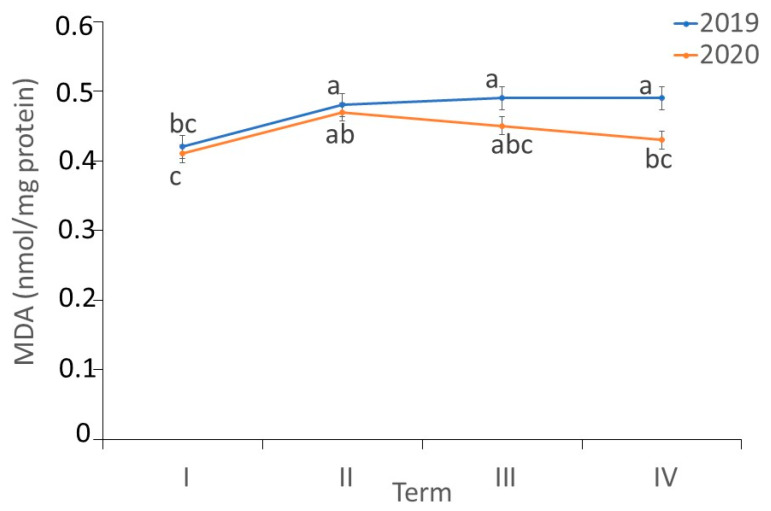
Changes in the malondialdehyde (MDA) content (nmol/mg protein) in *A. fabae fundatrigeniae* during feeding on *V. opulus* (means ± SD; *n* = 4). Different letters denote significant differences (one–way ANOVA; Tukey’s test; *p* < 0.05).

There were differences in the content of MDA in the *fundatrigeniae* of *A. fabae* during feeding on *V. opulus* (ANOVA, F_7,24_ = 9.97, *p* < 0.001). The MDA concentration in *fundatrigeniae* tissues was almost invariable during their feeding on *V. opulus*; only at the first date of observation in 2019 was it lower compared to the others. In most of the terms of the experiment, no differences in MDA content in the wingless females were noted during the studied years. At the decline of the aphid population on viburnum, a higher MDA content in *fundatrigeniae* was recorded in 2019 (Figure 6).

### 3.5. Changes in SOD, CAT and APX Activities in the Fundatrigeniae of A. fabae during Their Occurrence on the Primary Host

**Figure 7:**
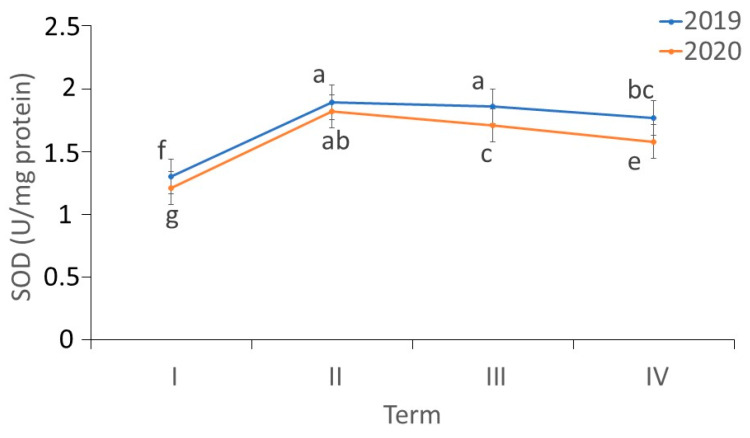
Changes in the SOD activity (U/mg protein) in *A. fabae fundatrigeniae* during feeding on *V. opulus* (means ± SD; *n* = 4). Different letters denote significant differences (one–way ANOVA; Tukey’s test; *p* < 0.05).

There were differences in the activity of SOD in the *fundatrigeniae* of *A. fabae* during feeding on *V. opulus* (ANOVA, F_7,24_ = 269.46, *p* < 0.001). The lowest SOD activity in the *fundatrigeniae* was noted in the first period of aphid development on the primary host. While the aphid population increased, a rapid increase in SOD activity was observed in the tissues of the studied morphs. In 2019, the induction of SOD activity persisted until the beginning of June, while in 2020, enzyme activity decreased during this period. However, SOD activity in the last generation of *fundatrigeniae* was still higher compared to the first morphs occurring on the primary host. In the first, third and fourth terms of observation, a higher SOD activity in *fundatrigeniae* was observed in 2019 (Figure 7).

**Figure 8:**
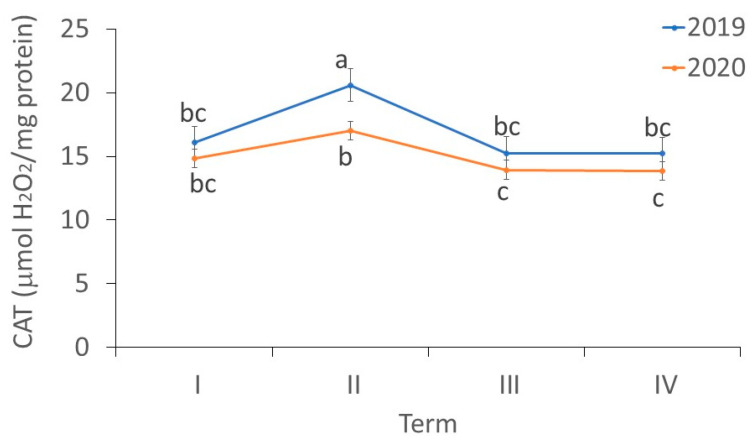
Changes in the CAT activity (µmol H_2_O_2_/mg protein) in *A. fabae fundatrigeniae* during feeding on *V. opulus* (means ± SD; *n* = 4). Different letters denote significant differences (one–way ANOVA; Tukey’s test; *p* < 0.05).

There were differences in the activity of CAT in the *fundatrigeniae* of *A. fabae* during feeding on *V. opulus* (ANOVA, F_7,24_ = 9.07, *p* < 0.001). The activities of CAT in *fundatrigeniae* tissues were quite similar in the first, third and fourth observation periods in both examined years. In 2019, the highest CAT activity in females was noted in the period with the largest density of aphids on viburnum. However, CAT activities in *fundatrigeniae* collected in 2020 were comparable at the beginning of the occurrence of insects and at their peak abundance on the primary host. No differences were found in CAT activity in female tissues between the studied years, with the exception of the second term of the experiment, when *fundatrigeniae* collected in 2019 were characterized by higher CAT activity (Figure 8).

**Figure 9:**
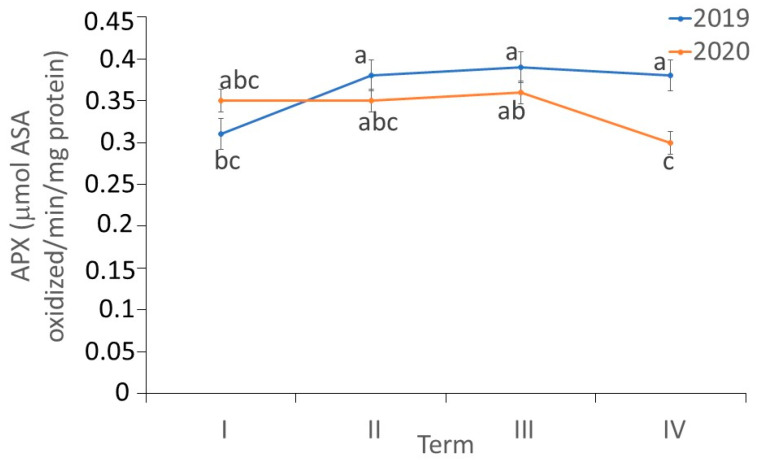
Changes in the APX activity (µmol ASA oxidized/min/mg protein) in *A. fabae fundatrigeniae* during feeding on *V. opulus* (means ± SD; *n* = 4). Different letters denote significant differences (one–way ANOVA; Tukey’s test; *p* < 0.05).

There were differences in the activity of APX in the *fundatrigeniae* of *A. fabae* during feeding on *V. opulus* (ANOVA, F7,24 = 7.21, *p* < 0.001). The APX activity in *fundatrigeniae* was comparable on most observation dates in both studied years. In 2019, a significant increase in APX activity in *fundatrigeniae* was observed at the peak of aphid abundance, and the induction was maintained until the end of the experiment. In 2020, lower APX activity was recorded at the decline of the aphid population on *V. opulus*, but it was still comparable to the first and second periods of observation. The APX activity in aphid tissues was similar in both studied years, except for the second decade of June, when higher activity was found in 2019 (Figure 9).

## 4. Discussion

During the ontogenesis in the plant leaves, an increase in dry mass and a decrease in the content of basic nutrients is observed. This phenomenon is extremely non-profitable for the aphids and it limits the further growth and development of the aphid population on hosts [10,11]. In our research, the first individuals of *A. fabae* on *V. opulus* were found in the second decade of April and disappeared in the third decade of June. These observations were made under natural conditions, so the aphids were exposed to the influence of weather conditions, as well as their natural enemies. Heavy rainfall and low temperatures lead to a slowdown or inhibition of the reproductive processes of aphids, and thus, the number and density on the host plants decreases [39,40]. The analysis of the population structure of *A. fabae* on the *V. opulus* in the studied years showed the lowest percentage share in the population for *fundatrices*. Population studies show that the initial size of the *fundatrices* generations is one of the factors that influences the population size of oligophagous aphids on the primary hosts [41]. Our observations showed that *A. fabae fundatrigeniae* on the primary host were the dominant morphs on the primary host, demonstrating the dynamic development and high reproductive potential of the aphid population. A major role of *fundatrigeniae* in the development of the oligophagous population on the primary hosts is the building of a population. Other functions relate to the exploitation of the host habitat, leading to a decrease in the content of major nutritive compounds. These physiological, biochemical and metabolic disturbances occurring within host tissues in response to aphid feeding may stimulate the production of *alatae*, rejection of host plants, and migration onto secondary, more suitable hosts [42]. In the period when the number of *fundatrigeniae* was at its maximum, the first winged morphs were found. They showed a weaker relationship with the primary host. They have a well-developed sensorimotor apparatus that enables them to actively search for a host. The production of these morphs is conditioned by food quality, in addition to environmental factors and population density [43,44,45]. The quantity and proportion of chemical compounds in consumed food are also important factors for aphid behavior, growth and development [46,47]. Several examples of chemicals can be found in the literature in guelder rose, which is the primary host of *A. fabae* [48,49,50,51,52], but their effect on this species has not been extensively studied. Primary host leaves possessed a high nutritive value of food for non-winged morphs that was not sufficient for *alatae*. Changes in the free amino acid content may stimulate rejection of the primary host and migration onto secondary plant hosts [10,53]. Plant phenolic compounds can affect the performance, fecundity and survival of herbivores [11,54,55].

The overproduction of ROS in host plants negatively affects herbivores by evoking oxidative stress in their tissues. Our experiments showed that the feeding of *A. fabae* on viburnum plants was associated with ROS generation ($O_{2^{-}}$ and H_2_O_2_). Little is known about ROS generation in aphids. However, the presence of these molecules has been found in the tissues of cereal aphids [56,57,58,59], the pea aphid *Acyrtosiphon pisum* (Harris) [60,61] and the green peach aphid *Myzus persicae* (Sulzer) [62]. Our data demonstrate clear differences in ROS content between various morphs of *A. fabae* occurring on the primary host. The lowest $O_{2^{-}}$ and H_2_O_2_ content were recorded for the winged migrants. The opposite trend was observed in cereal aphids fed on winter wheat, where *alatae* were characterized by the highest H_2_O_2_ content [58]. Additionally, winged adults of *A. fabae* that occurred on the primary host had a 2–3-fold lower H_2_O_2_ content than migrants of cereal aphids infesting winter wheat plants [58]. This may be related to the more efficient antioxidant mechanisms of winged *A. fabae* females.

ROS accumulation in cells may cause alterations in the structures of proteins, lipids and DNA. One of the final products of lipid peroxidation is MDA, which can damage proteins by addition reactions with lysine amino groups, cysteine thiol groups and histidine imidazole groups [63]. There are not many reports concerning lipid peroxidation in sucking-piercing insects. In the current work, the lowest MDA content was recorded for winged adults, while wingless morphs (*fundatrices* and *fundatrigeniae*) had a similar MDA concentration. This is probably associated with a lower content of ROS in migrant tissues in comparison to wingless females. Other results were obtained by Łukasik et al. [58], where the *alatae* morphs of *Sitobion avenae* (F.) and *R. padi* were characterized by higher MDA content than *larvae* and *apterae*. Moreover, the migrants of cereal aphids feeding on winter wheat had almost 7-fold higher MDA levels than the winged morphs of black bean aphids that occurred on viburnum [58]. Thus, the low H_2_O_2_ and MDA content in migrants of *A. fabae* points to their poor association with the primary host and specialization towards successful colonization of the secondary host.

Aphids developed deceptive strategies that enabled them to avoid plant defenses. Aphid saliva introduced into plant tissues contains effectors that affect the defense-signaling pathways triggered by plants [64]. In response to oxidative stress arising from host plants, aphids evolved antioxidant systems removing ROS consisting of SOD, CAT and APX. The highest activity of antioxidant enzymes was noted for winged adults of *A. fabae*, which agrees with the results obtained for *R. padi* feeding on bird cherry [30]. High levels of antioxidant enzymes in migrant tissues appear to be a form of adaptation to the colonization of new host plants. The winged *A. fabae* females had a lower SOD content but higher CAT activity than winged migrants of *R. padi* feeding on bird cherry (*Prunus padus* L.) leaves [31]. However, cereal aphids infesting winter wheat exhibited higher CAT and APX activity than *A. fabae* morphs [28,65]. This could be a consequence of a higher H_2_O_2_ concentration in the tissues of cereal aphids. SOD and CAT activities in *A. fabae* morphs were lower than those reported by Durak et al. [66] for aphid *Cinara (Cupressobium) tujafilina* (Del Guercio 1909) feeding on *Thuja orientalis* L. plants. This may be associated with differences in the chemical composition of plants that are hosts for these aphid species. Additionally, *C. tujafilina* was reared in a climate chamber, while *A. fabae* morphs were fed in a natural environment. CAT and APX activities in *M. persicae* were much higher than those found in *A. fabae* morphs [67]. This is because the studied host plants of *M. persicae* (cumin, anise and coriander) are rich in pro-oxidant furanocoumarins and β-carboline alkaloids [68].

Changes in the quality of primary host plants affect the development and metabolic processes of aphids. *Fundatrigeniae* of *A. fabae* are morphs that occur on the primary host for the longest time and seem to be more closely associated with it than migrants. We observed a significant enhancement of the ROS and MDA content in the tissues of *fundatrigeniae* in the period when the density of the *A. fabae* population on viburnum rapidly increased. Such induction may be related to changes in the quantitative composition and pro-oxidant content of the host plant. Czerniewicz et al. [53] stated that the total phenolic content in bird cherry leaves increased with the development of the *R. padi* population. The authors noted the highest concentrations of phenolic compounds in *P. padus* tissues just before the spring migration of *R. padi*. The results of our earlier studies showed that the exposure of cereal aphids to o-dihydroxyphenols caused the accumulation of H_2_O_2_ and lipid peroxidation products in their tissues [58]. Moreover, treatment with essential oils induced ROS generation and damage to lipids within the tissues of *M. persicae* and *R. padi* [62]. The host plants and their specific chemical compositions can affect the oxidative balance of aphids. $O_{2^{-}}$ and H_2_O_2_ content increased when the cereal aphids were transferred from winter wheat to winter triticale and the increase was greater for the less susceptible cultivar ‘Witon’ in comparison to the more susceptible cultivar ‘Tornado’ [59]. In addition to plant allelochemicals, temperature can generate ROS and cause oxidative stress in aphid tissues. Khursid et al. [69] showed that heat stress significantly increased the H_2_O_2_ and MDA content in *M. persicae*.

Similar to oxidative stress markers, we noted the induction of antioxidant enzyme activities within *fundatrigeniae* tissues just before the spring migration of *A. fabae*. This agrees with the results obtained by Leszczyński et al. [70] for the *R. padi* population occurring on bird cherry. The growth of SOD and CAT activity in the *fundatrigeniae* of *A. fabae* may be a response to ROS elevation within their tissues during the peak of aphid abundance in the primary host. In response to rising aphid population size, host plants may intensify allelochemical synthesis and alter antioxidant enzyme activity. Previous reports indicated that SOD, CAT and APX activity increased when cereal aphids were exposed to o-dihydroxyphenols [28,65,71]. Czerniewicz and Chrzanowski [62] demonstrated that treatment with essential oils composed of secondary plant metabolites led to a significant upregulation of SOD and CAT activity in *R. padi* and *M. persicae*. The authors state that the mode of action of these oils may be related to the generation of oxidative stress within aphid tissues. Rup et al. [72] observed that the SOD and CAT activities in the kinetin-treated nymphs of the mustard aphid *Liphaphis erysimi* (Kalt.) were significantly higher than under normal developmental conditions. However, CAT activity in *S. avenae* was strongly suppressed by the higher concentration of catechol, gramine and L-ornithine HCl, whereas a lower concentration of gramine stimulated CAT activity [73]. Therefore, the authors indicated that the titer of plant allelochemicals in host plants may not be sufficient to inhibit CAT activity in herbivores. Additionally, host plants can affect antioxidant enzyme activity. Abdelsalam et al. [67] showed that the studied host plants (cumin, anise and coriander) clearly affected the antioxidant enzyme activity in *M. persicae*. SOD and CAT activities increased soon after the winged adults of *R. padi* originating from bird cherry were transferred to the secondary host spring triticale [31]. A similar tendency was observed for SOD, CAT and APX activities of cereal aphids when insects were transferred from winter wheat to winter triticale [59]. Durak et al. [66] demonstrated that switching the host plant stimulated SOD and CAT activities in the tissues of *C. tujafilina*. *A. pisum* adults reared on broad bean plants had 3-fold higher antioxidant enzyme activity than those fed on pea and vetch [29]. Thus, the antioxidant mechanisms of aphid seem to be flexible and are a type of adaptation to various host plants. The antioxidant defense system of aphids is also influenced by temperature. Durak et al. [74] and Dampc et al. [75] showed that an increase in temperature caused alterations in SOD and CAT activity, which were highest at 28 °C, in *Aphis pomi* (De Geer), *Macrosiphum rosae* (L.) and *Cinara cupressi* (Buckton). Khursid et al. [69] stated that SOD and CAT activity in *M. persicae* adults increased under short-term heat stress.

## 5. Conclusions

In summary, the results presented here demonstrate that feeding *A. fabae* morphs to the primary host affects the redox balance within their tissues. Low levels of oxidative stress markers combined with high antioxidant enzyme activity in the tissues of migrants suggest that winged females are specialized to switch between primary and secondary host plants.

## Data Availability

The data presented in this study are available in the article.
